# Identification of copy number variation in French dairy and beef breeds using next-generation sequencing

**DOI:** 10.1186/s12711-017-0352-z

**Published:** 2017-10-24

**Authors:** Rabia Letaief, Emmanuelle Rebours, Cécile Grohs, Cédric Meersseman, Sébastien Fritz, Lidwine Trouilh, Diane Esquerré, Johanna Barbieri, Christophe Klopp, Romain Philippe, Véronique Blanquet, Didier Boichard, Dominique Rocha, Mekki Boussaha

**Affiliations:** 1 GABI, INRA, AgroParisTech, Université Paris-Saclay, 78352 Jouy-en-Josas, France; 2 GMA, INRA, Université de Limoges, UMR1061, Unité Génétique Moléculaire Animale, 123 avenue Albert Thomas, 87060 Limoges Cedex, France; 3 Allice, Maison Nationale des Eleveurs, 75012 Paris, France; 4 LISBP, CNRS, INRA, INSA, Université de Toulouse, Toulouse, France; 5 GenPhySE, INRA, Université de Toulouse INPT ENSAT, Université de Toulouse INPT ENVT, 52627 Castanet-Tolosan, France; 6 SIGENAE, INRA, 52627 Castanet-Tolosan, France

## Abstract

**Background:**

Copy number variations (CNV) are known to play a major role in genetic variability and disease pathogenesis in several species including cattle. In this study, we report the identification and characterization of CNV in eight French beef and dairy breeds using whole-genome sequence data from 200 animals. Bioinformatics analyses to search for CNV were carried out using four different but complementary tools and we validated a subset of the CNV by both in silico and experimental approaches.

**Results:**

We report the identification and localization of 4178 putative deletion-only, duplication-only and CNV regions, which cover 6% of the bovine autosomal genome; they were validated by two in silico approaches and/or experimentally validated using array-based comparative genomic hybridization and single nucleotide polymorphism genotyping arrays. The size of these variants ranged from 334 bp to 7.7 Mb, with an average size of ~ 54 kb. Of these 4178 variants, 3940 were deletions, 67 were duplications and 171 corresponded to both deletions and duplications, which were defined as potential CNV regions. Gene content analysis revealed that, among these variants, 1100 deletions and duplications encompassed 1803 known genes, which affect a wide spectrum of molecular functions, and 1095 overlapped with known QTL regions.

**Conclusions:**

Our study is a large-scale survey of CNV in eight French dairy and beef breeds. These CNV will be useful to study the link between genetic variability and economically important traits, and to improve our knowledge on the genomic architecture of cattle.

**Electronic supplementary material:**

The online version of this article (doi:10.1186/s12711-017-0352-z) contains supplementary material, which is available to authorized users.

## Background

For the first time in 2004, copy number variations (CNV) were reported as a new form of genomic alteration [1, 2]. CNV are defined as gains or losses of DNA segments ranging from 50 bp to several megabases (Mb). CNV are considered to be polymorphic genetic markers and are inherited across generations [3]. At the genome level, CNV are less frequent than single nucleotide polymorphisms (SNPs) and small insertions and deletions (InDel), but they can have a greater functional and evolutionary impact. For example, by modifying the genome organization, CNV can affect gene expression and therefore certain phenotypes of interest [4].

CNV and their impact have been extensively studied in several species, particularly in humans, in which they are known to cause several genetic diseases. For example, a 2-kb deletion located upstream of the *IRGM* (*immunity related GTPase M*) gene is linked with Crohn’s disease [5], a CNV located within the *TSPAN8* (*tetraspanin 8)* gene is associated with type 2 diabetes [6], and a duplication within the *CCL3L1* (*C*–*C motif chemokine ligand 3 like 1*) gene is involved in HIV susceptibility [7].

In domesticated animals, CNV are also linked with several phenotypic traits. For example, two duplications that overlap with the *KIT* (*KIT proto*-*oncogene receptor tyrosine kinase*) and *ASIP* (*agouti signaling protein*) genes are responsible for white coat color in pigs and sheep, respectively [8, 9]. In chickens, the pea-comb phenotype is associated with a duplication within the *SOX5* (*SRY*-*box 5*) gene [10, 11]. In ridgeback dogs, a 133-kb duplication is located within the genomic region that contains the *FGF3* (*fibroblast growth factor 3*), *FGF4* (*fibroblast growth factor 4*), *FGF19* (*fibroblast growth factor 19*), and *ORAOV1* (*oral cancer overexpressed 1*) genes and causes both hair ridge and a predisposition to dermoid sinus [12]. In cattle, anhidrotic ectodermal dysplasia is induced by a deletion in the *ED1* (*anhidrotic ectodermal dysplasia*) gene [13], and polled and multisystemic syndrome is caused by a deletion that knocks out the *ZEB2* (*zinc finger E*-*box binding homeobox 2*) gene [14].

Both array-based comparative genomic hybridization (CGH) and SNP arrays have long been widely used for the detection of CNV. However, these two approaches are not very efficient and lack the sensitivity needed to detect a wide range of CNV [15–18]. For example, the resolution of the array-based CGH approach depends on the number, size, and quality of the probes fixed on the array [15–18]. Thus, with a low-density array, it is difficult to detect all the small variants. Similarly, detection of CNV with SNP genotyping arrays depends on the nature of the SNPs included and their distribution within the genome and if the density of the SNP array is low, detection of small variants is unlikely.

The advent of whole-genome sequencing, coupled with major bioinformatics developments, have profoundly modified strategies used to detect CNV. Unlike array-based CGH and SNP array platforms, the next-generation sequencing (NGS)-based approach can identify a wide range of CNV, ranging in size from tens of nucleotides to several Mb, with accurate localization of breakpoints.

In this study, we performed a genome-wide characterization of CNV in cattle using four software packages based on three different approaches. We performed a bioinformatics search for CNV by exploring whole-genome sequencing data from 200 animals that represented eight French dairy and beef breeds.

## Methods

### Animal ethics

Most whole-genome sequences used in this study were already available in our laboratory (see [19] for more details), and thus no animal experimentation was necessary for this study. A small part of our dataset was generated from 23 genomic DNA samples that were obtained from muscle tissue collected at commercial slaughterhouses. Five other genomic DNA samples were prepared from sperm collected from semen straws that were provided by approved commercial artificial insemination stations as part of their regular semen collection process.

### Genomic DNA extraction and whole-genome sequencing

Details on the extraction of genomic DNA for 172 of the animals are in [19] and DNA extraction for the remaining 28 animals was performed using the Wizard Genomic DNA Purification kit (Promega, Charbonnières-les-Bains, France). Each purified DNA sample was quality-controlled by agarose gel electrophoresis. DNA concentration was then measured with a Nanodrop ND-100 instrument (Thermo Fisher Scientific, Ilkirch, France). Genomic DNA library construction and sequencing for the 200 animals were performed as previously described [20]. All sequences were then aligned to the UMD3.1 reference genome sequence with the Burrows-Wheeler aligner (BWA) [21].

### DNA sampling

Two hundred French cattle were selected for sequencing as representative of four main dairy, i.e. Brown Swiss, Holstein, Montbéliarde, and Normande and four main beef breeds, i.e. Blonde d’Aquitaine, Charolaise, Limousine, and Rouge des Prés (Table 1) and Table S1 (see Additional file 1: Table S1). Of these 200 animals, 14 were sequenced at least twice. In addition, two sire-dam-son trios (both Montbéliarde) and 16 sire-son pairs (six Normande, eight Montbéliarde, and two Holstein pairs) were included.

**Table 1:** Distribution of animals per breed and sequencing coverage

Breed	Number of animals	Coverage min–max	Coverage mean	Percentage of chimeric reads	Breed type
Blonde d’Aquitaine	25	11–26	15	1.1 ± 0.1	Beef
Brown Swiss	3	9–12	10	0.3 ± 0.03	Dairy
Charolaise	25	11–25	15	1.3 ± 0.2	Beef
Holstein	56	8–21	13	0.8 ± 0.9	Dairy
Limousine	34	8–25	14	1.6 ± 0.8	Beef
Montbéliarde	31	9–28	15	1.6 ± 1.1	Dairy
Normande	23	8–33	12	2.3 ± 0.9	Dairy
Rouge des Prés	3	16–31	21	1.3 ± 0.1	Beef
Total	200	8–33	14.4	1.4 ± 1.5	

### Detection of CNV

Computational approaches for searching CNV in whole-genome sequence data involved four commonly used tools. CNVnator v0.3 [22] identified CNV using a read-depth (RD) approach within genomic windows of 250 bp. BreakDancer v1.3.6 [23] was run with default parameters to detect CNV with the paired-end mapping (PEM) approach. Both Pindel v2.5 [24] and DELLY v0.6.1 [25] software packages use a PEM-based strategy followed by a split-read (SR)-based approach to determine the type and the size of the predicted variant. Pindel and DELLY were used with default parameters.

### Analysis of CNV

**Fig. 1:**
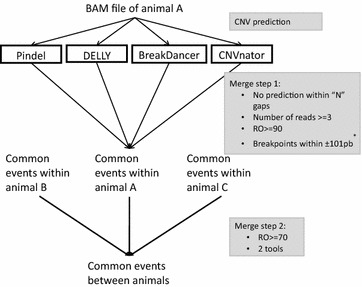
Flowchart of pipeline used to identify copy number variations (CNV). *RO* reciprocal overlap. *Only for the comparison of CNV between Pindel and DELLY. First, CNV were predicted separately by each tool within each sample. Second, only predictions supported by at least three reads and covered by less than 25% of their sequence of “N” gaps were retained. These filtered CNV were compared within the same sample to retain common CNV predicted by the different tools. CNV should share at least 90% of their sequence between two tools. Finally, CNV were identified by merging variants at 70% of reciprocal overlap across all samples

For each animal, first we excluded all variants for which the breakpoint positions were located within a 100-bp window that contained a gap in the reference sequence. Then, we filtered out all variants for which more than 25% of the bases consisted of gaps. Information about the location of all unknown sequences within the UMD3.1 reference genome sequence was downloaded from the NCBI database (ftp://ftp.ncbi.nlm.nih.gov/genomes/Bos_taurus/Assembled_chromosomes/agp/). Finally, we selected all variants that were supported by a minimum of three reads and retained only those that were predicted by at least two different tools. In addition, a variant that was predicted by both Pindel and DELLY was retained only if these two methods identified the corresponding breakpoint positions that were within 100 bp of each other (i.e. the 5′ breakpoint indicated by Pindel was within 100 bp of the breakpoint identified by DELLY, and the same for the 3′ breakpoint). For all other combinations of tools, we applied a 90% reciprocal overlap (RO) threshold for defining CNV as belonging to the same region; otherwise, they were considered as distinct regions (Fig. 1).

Variants that passed these filtering criteria were subsequently checked in other samples. A given CNV was defined as common to at least two samples when the predicted region in one sample had at least 70% reciprocal overlap with the CNV region predicted in another sample. The resulting overlapping variants were then used to define potential CNV regions (CNVR). The new breakpoint positions and the size of these CNVR were defined as follows: (1) the 5′ and 3′ genomic positions of the CNVR corresponded respectively to the lowest 5′ and the highest 3′ positions of all overlapping variants identified in the previous steps; and (2) the size of the CNVR was defined as the interval between the new 5′ and 3′ breakpoint positions.

### Validation of CNV

#### Mendelian approach

CNV were validated by two in silico and two experimental approaches. First, two family trios and 16 sire-son pairs from our dataset were sequenced. Since CNV should be inherited from parent to son, we reported only CNV that were present in either of the parents and the offspring in trios or in both sire and son in sire-son pairs.

#### Twice-sequenced approach

Second, we used 14 animals for which we had sequencing data generated from at least two different sequencing runs in order to estimate the number of shared predicted CNV between the two datasets. Theoretically, for a given animal, the same CNV should be present in each of the two independent sequences.

#### Array-based CGH approach

The third validation approach involved array-based comparative genomic hybridization (CGH) analysis. In this study, CGH experiments were performed using the Agilent CGH array (SurePrint G3 Bovine CGH Microarray, 4 × 180 K), which contained 152,934 oligonucleotide probes, each 45 to 60 nucleotides long. Adjacent probes were on average 16,376 bp apart on the UMD3.1 reference genome. DNA for 17 animals was analyzed using this CGH microarray, including DNA from Dominette (the animal that was used to generate the bovine reference genome), which was used for normalization steps. The 17 animals were also sequenced in this study (the detailed protocol for array-based CGH is available in Additional file 2). Briefly, 600 ng of fragmented DNA was labeled with Cy3- or Cy5-labeled nucleotides (tested DNA and control DNA, respectively). Tested and control DNA were then co-hybridized in the Agilent system and the array was scanned on the MS200 scanner (TECAN) according to the manufacturer’s instructions and as performed in other studies [26, 27]. Fluorescence intensities were normalized and quality was checked using the Feature Extraction software v11.5.1.1 from Agilent. Then, the aberration detection method 2 (ADM-2) algorithm was applied using Agilent Genomic Workbench software (v7.0.4.0) to detect variants. Potential variants were detected by analyzing aberrations in the normalized fluorescence intensities relative to the reference DNA sample, Dominette (log 2 ratio). A CNV was retained if at least three consecutive probes supported it. Finally, CNVR were defined by comparing identified CNV across samples. Two CNV belonged to the same CNVR if they shared at least 50% of their sequence.

#### Custom SNP genotyping approach

Finally, we selected 122 deletion-only, duplication-only, or CNV regions for testing in genotyping assays using the Illumina bovine low-density BeadChip (Infinium^®^ BovineLD v6.0: LDv6) [28]. In this chip, each variant was represented by at least three SNPs that were uniformly spaced. These SNPs were chosen from the SNP catalog published by Boussaha et al. [19]. Overall, 1008 SNPs were genotyped for 14,082 animals from 18 different breeds by LABOGENA SA (France), following the manufacturer’s recommendations. Total signal intensity (Log R Ratio: LRR) and allelic intensity ratio (B allele frequency: BAF) of the SNPs for each sample were collected and then analyzed with PennCNV (2011Jun16 version) [29] to identify CNV. Only samples with an LRR standard deviation less than 0.3 and a BAF drift less than 0.01 were considered for detection. Then, the resulting variants were compared to those predicted with WGS data. As for the array-based CGH approach, two variants were considered as identical if at least 50% of their sequence were shared and if they passed all other in silico approaches.

#### Statistics

All reported statistics were calculated using R software. The Pearson method was used first to assess the correlation between reported variants and coverage rate, and second to calculate the correlation between the number of variants and chromosome size. The Chi square test was used to evaluate within-breed genetic variability. This within-breed genetic variability was assessed by comparing the number of predicted variants to the expected number, which was calculated as the ratio between number of predicted variants and number of sequenced animals within each breed.

### Comparison of CNVR with known bovine CNV

We compared our CNV dataset with those previously published for cattle [4, 20, 30–34] and with the publicly available Genomic Variants archive (DGVa) database of EMBL-EBI (http://www.ebi.ac.uk/dgva). These publicly available CNV were detected using array-based CGH [30], SNP genotyping arrays [31, 32], and whole-genome sequencing [4, 20, 33, 34]. The comparison was carried out using the Bedtools software package [35]. Given the differences between platforms, definitions of CNV, and methods of CNVR construction used, two CNV were considered as shared when there was at least 50% of reciprocal overlap between the two regions.

### Gene content and gene ontology

First, functional elements that were located within or overlapping with deletion-only, duplication-only, and CNV regions were identified by using a custom python script (available upon request) that was coupled with the “intersectBed” option of the Bedtools package [35]. Gene content was analyzed by using the bovine Ensembl genebuild database (version 89) retrieved from the BioMart database (http://www.ensembl.org/biomart/). In total, 24,616 bovine genes were downloaded. For each gene that was located within a CNVR or overlapping with a CNVR, we used Ensembl information to verify whether there were known paralogous genes.

Second, we used the public animal QTLdb database, release 32 [36], to check whether the variants included in our panel were located within or overlapped with publicly available bovine quantitative trait loci (QTL).

Finally, gene ontology (GO) and Kyoto Encyclopedia of Genes and Genomes (KEGG) pathway analyses were performed using the PANTHER classification system v11.1 [37]. After Bonferroni correction, enriched GO terms within biological processes, cellular components, and molecular functions were identified.

## Results and discussion

### Sequencing data

One hundred seventy-two French beef and dairy animals were sequenced as described by Boussaha et al. [19] (see Additional file 1: Table S1). Sequencing details for the 28 whole-genome sequences that were obtained for this study are in Table S1 (Additional file 1: Table S1). As in Hoze et al. [38], 180 sires of the 200 sequenced animals were chosen based on their marginal contribution to their population based on pedigree information, as defined by Boichard et al. [39], and computed using the PEDIG software [40]. Eighteen of the remaining 20 animals were sons of some of these 180 selected sires. In addition, two mothers of some of the 18 sons were chosen.

Paired-end sequencing produced 71.51 billion paired-ends read, of which 69.46 billion (96.5%) were correctly mapped to the UMD3.1 reference genome sequence. The average insert size was 321 bp and the average whole-genome sequencing coverage was 14.4x, ranging from 8× to 33× (see Additional file 1: Table S1).

### Deletion, duplication, and CNV calls

Deletions, duplications, and CNV regions were predicted using four tools. To define potential CNVR, raw events predicted by all four tools were merged and further analyzed (Fig. 1). Overall, we detected 19,077 deletion and duplication events predicted by at least two tools (see Additional file 3: Table S2).

### Validation of CNVR

Given the huge number of detected variants and the likelihood of a relatively high rate of false positives due to the number of approaches used, we decided to apply multiple criteria to retain the most reliable variants. Thus, all detected variants were validated by applying four strategies, in silico and experimentally, and only the validated ones were considered for further analyses.

#### Mendelian approach

**Fig. 2:**
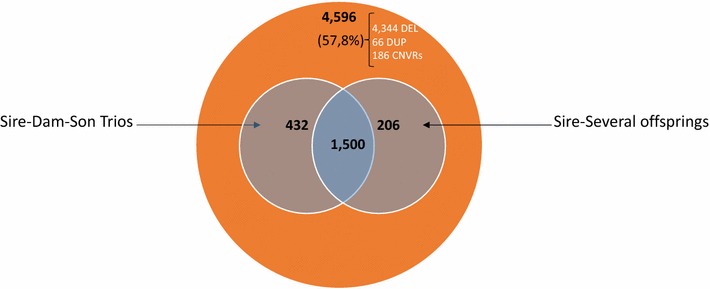
Number of variants supported by Mendelian inheritance. The total number of variants supported by Mendelian inheritance is indicated in the orange circle (two family trios and 16 sire-son pairs). The sire-dam-son trios legend represents the number of variants found in the offspring and in its parents. Sire-several offspring indicates the number of variants that were predicted for the sire and several of its offspring

Analysis of trios and parent–offspring pairs revealed that 57% (4596 of 8088) of the identified variants were present in at least one parent and one of its offspring (Fig. 2), Table S1 (see Additional file 1: Table S1) and Table S3 (see Additional file 4: Table S3). Of these 4596 variants, almost 33% were present in all three members of the trio (sire, dam, and offspring) and in all offspring of a given sire (in cases in which a sire had several offspring). The percentage of variants that were transmitted from either of the parents to the offspring was highly variable, ranging from 12 to 72%, and was highly correlated with sequencing depth (Pearson correlation score *ρ* = 0.75, *p* value = 1.6e−4) (see Additional file 5: Table S4).

#### Twice-sequenced approach

Next, we compared the variants detected in whole-genome sequence data generated from two or more sequencing runs of the same individual. In total, 7394 of the 9266 variants that were predicted for these 14 animals were found in both sets of sequence data by at least one CNV detection tool (see Additional file 6: Table S5). The overall concordance rate was around 80%. Of these 7394 variants, 44% (3261) were validated in at least two different breeds, 38% were validated only in Montbéliarde, 4% only in Charolaise, 6% only in Limousine, 4% only in Rouge des Prés, and 2% only in Blonde d’Aquitaine. Moreover, around 56% (4163) of these variants were also confirmed by the Mendelian strategy.

#### Array-based CGH approach

Array-based CGH analysis of whole-genome sequence data from 17 individuals resulted in the identification of 68 variants (see Additional file 7: Table S6). The detection of a given type of variants for one animal with the array-based CGH method was compared to that with the whole-genome sequence method (reciprocal overlap (RO) ≥ 50%); of these 68 variants, 34% (23 variants) were found by both approaches. In addition, when variants detected by array-based CGH were compared with raw variants data that were predicted using whole-genome sequence data prior to merging, we retrieved 18% (12 variants) more than in the analysis that used only merged variants. These 12 variants were mostly predicted with CNVnator (92%, 11 variants) (see Additional file 7: Table S6). Following this comparison, 22% (15 variants) of the remaining variants detected by array-based CGH were found in the whole-genome sequence dataset if we relaxed the RO threshold to 1%; the fact that these variants were excluded from the sequence approach showed the stringency of the criteria that we used to define variants (see Additional file 7: Table S6). The remaining 26% (18 variants) of the variants detected by array-based CGH not found with whole-genome sequence data were located in poorly sequenced regions. Among the 23 variants detected by both array-based CGH and whole-genome sequence approaches, eight were also found in animals sequenced in at least two sequencing runs, and three were also validated by the Mendelian approach.

### Custom SNP genotyping approach

Finally, we used SNP genotyping assays to test 122 variants, which were found by the Mendelian and twice-sequenced approaches. These variants were selected based on their frequency (≥ 10%) in at least one of the three main French dairy breeds (Montbéliarde, Normande and Holstein). For each variant, we selected at least three SNPs from a publicly available SNP dataset [19]. We validated 69 variants (56%) (see Additional file 8: Table S7). Eight additional variants were retained by applying a RO threshold of 20%. In total, 45 variants that were predicted from WGS data were not captured with the approach based on SNP genotyping data. These variants may have been excluded either during the filtering steps of the SNP genotyping quality control or during the CNV identification process, in which three SNPs were needed to retain the CNV.

Overall, we retained 4178 variants that were detected by both in silico approaches (Mendelian and twice-sequenced approaches) and/or CGH. Of these 4178 variants, 83% (3464) were predicted by a minimum of three tools and 22.2% (927) were predicted by all four tools (see Additional file 9: Table S8). Most validated variants were predicted by both DELLY and BreakDancer (4075 variants, 97.5%). The smallest number of validated variants was identified by the combination of Pindel + CNVnator (1047 variants, 25%) (Table 2).

**Table 2:** Percentage of validated variants per combination of tools

Combination of tools	Number of variants before validation	Number of variants after validation	Validation rate (%)
Pindel + DELLY	7510	3169	42.2
Pindel + CNVnator	2048	1047	51.1
Pindel + BreakDancer	7364	3161	42.9
DELLY + CNVnator	4840	2139	44.2
DELLY + BreakDancer	17,479	4075	23.3
CNVnator + BreakDancer	6060	2327	38.4
Total	19,077	4178	

Analysis of the distribution of the percentage of validated variants per combination of tools shows that the Pindel + CNVnator combination yielded the highest percentage (1047 validated variants out of 2048 total predicted variants, 51.1%) followed by the DELLY + CNVnator combination (44.2% i.e. 2139 validated variants out of 4840 total predicted variants). DELLY combined with BreakDancer predicted the largest number of variants but only 23.3% of these (4075 validated variants out of 17,479 total predicted variants) were validated, which indicates that these tools have a high rate of false positives. This is consistent with previous reports that showed that DELLY outperforms the other tools in terms of discovery, but has a high rate of false positives [41, 42]. In contrast, all combinations of tools that included Pindel had a higher rate of validated variants (42%), which reflects the high degree of accuracy of this tool for predicting variants, which is likely due to Pindel using the split-read approach on one-end anchor reads to identify, with high resolution, the breakpoint positions of a variant.

#### Distribution of variants

We retained 4178 variants that were validated by both in silico approaches and/or array-based CGH and, across all animals, they represented 6% (150 Mb) of the UMD3.1 cattle genome assembly. These variants comprised 3940 deletion-only regions, 67 duplication-only regions and 171 CNVR (Table 3).

**Table 3:** Number of predicted and validated variants

Type of variant	Number of variants
Deletions	3940
Duplications	67
CNVR (deletion + duplication)	171
Total	4178

**Fig. 3:**
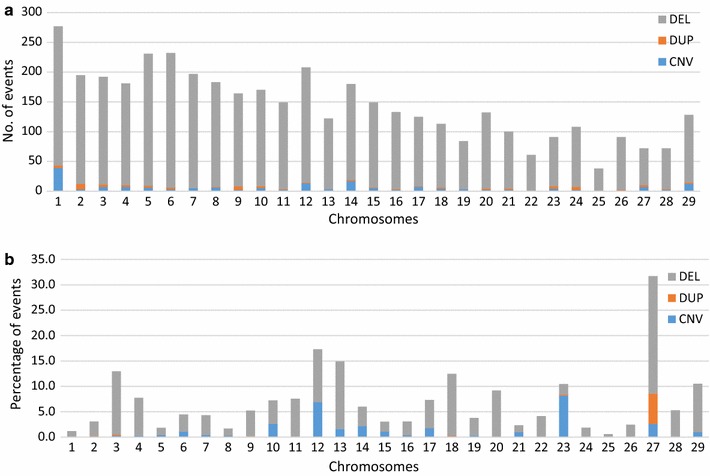
Distribution of variants on autosomal chromosomes. **a** Number of variants per chromosome. **b** Proportion of chromosome covered by variants. Deletions are represented by grey, duplications by orange and CNV regions by blue bars

Analysis of the distribution of variants on the autosomes revealed a significant correlation between the number of predicted variants and chromosome size (Pearson correlation score *ρ* = 0.91, *p* value = 5.56e−12, Fig. 3a). *Bos taurus* chromosome 1 (BTA1), 6, and 5 carried the largest number of variants (277, 232, and 231, respectively; (see Additional file 10: Table S9), whereas BTA25 had the smallest number (38). However, the correlations between number of predicted variants and proportion of each chromosome covered by variants were quite different (Fig. 3b). The highest percentage of sequence covered by CNV (30.2%) was found for BTA27, which was also the chromosome that was most covered by duplication-only regions (2.7 Mb, CNVR_11771). On the contrary, BTA3, 13 and 27 were the chromosomes that were most enriched in deletion-only regions (15.2, 11.2 and 10.5 Mb, respectively). BTA3 and 13 carried the largest deletions that we detected in our dataset i.e. 7.7 Mb (CNVR_13246) and 6.9 Mb (CNVR_3635), respectively. In addition, two large deletions (CNVR_11676 and CNVR_11898) that together covered 8.6 Mb were identified on BTA27. BTA12 and 23 carried the largest CNVR i.e. 6.3 Mb (CNVR_3252 and CNVR_3296) and 4.3 Mb (CNVR_10026), respectively.

In contrast, the lowest percentage of sequence covered by variants (0.6%) was found for BTA25 and for BTA1 (1.1%) although this chromosome had the largest number of CNV (see Additional file 10: Table S9). The total length of CNV per chromosome was not correlated with chromosome length (Pearson correlation score *ρ* = 0.12, *p* value = 0.54).

**Fig. 4:**
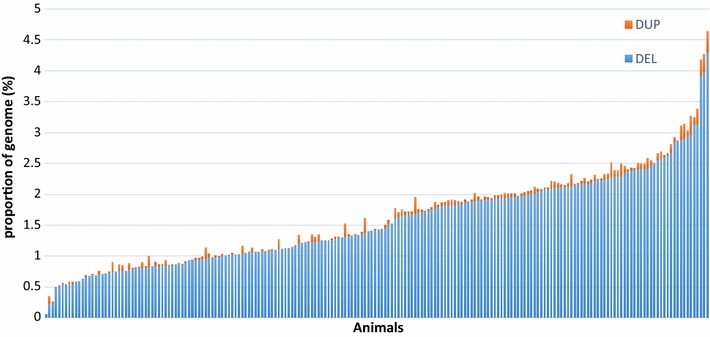
Proportion of variants in each sample. Blue bars indicates the proportion of deletions and orange bars indicate the proportion of duplications in each sample

**Fig. 5:**
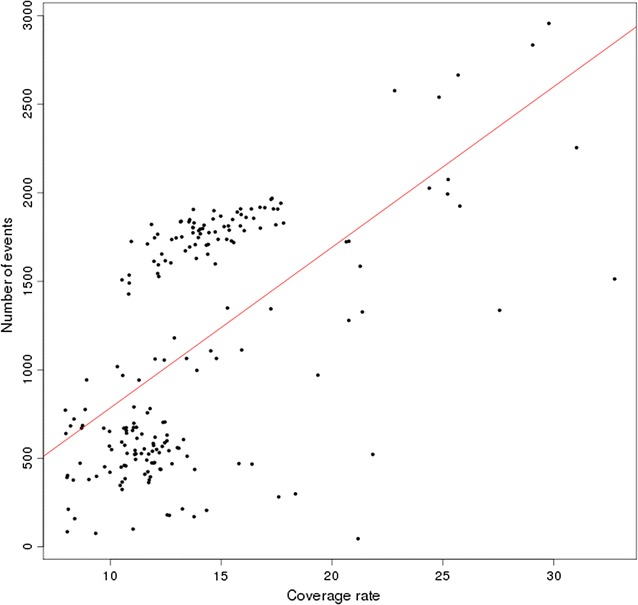
Link between the number of variants and the coverage rate for each animal

On average, we identified 1132 variants per individual and this number ranged from 46 to 2957. These variants covered 0.06 to 4.45% of the genome of each animal, with an average and median proportion of 1.6% ± 0.7 and 1.4%, respectively (see Additional file 1: Table S1) and see Fig. 4. This observed variability across individuals can be partly explained by variations in coverage depth, which ranged from 8× to 33× (Fig. 5). Indeed, the smallest number of detected deletions and duplications was found for animals with a low sequencing depth and this increased as sequencing depth increased (Pearson correlation score *ρ* = 0.60, *p* value < 2.2e−16).

**Fig. 6:**
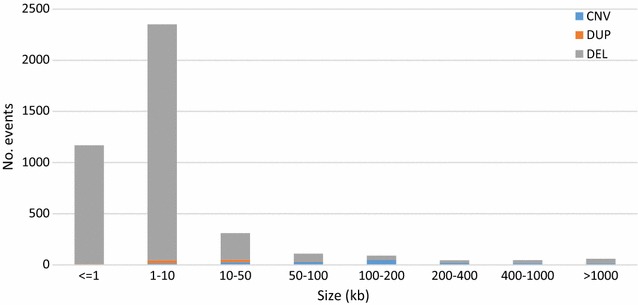
Distribution of the size of the deletions, duplications and CNVR

Analysis of the distribution of variant size revealed that 75% of the deletion-only regions were shorter than 3.3 kb with a median size of 1.5 kb (Fig. 6), whereas 75% of the duplication-only regions were longer than 4.4 kb, with a median size of 8.7 kb. Likewise, about 75% of CNVR were longer than 45.7 kb, with a median size of 114 kb (Fig. 6).

This finding confirms the results of a previous study in cattle [43] and may be explained by technical and/or biological factors. For example, our study only predicted tandem duplications because dispersed duplications are difficult to confirm by applying the combination of CNV detection tools that we used, thus the smaller number of detected duplications. In addition, some studies [44, 45] reported that certain recombination mechanisms, such as non-allelic homologous recombination (NHAR), result in more deletions than duplications, which may also partly explain the larger number of deletions observed in this work compared to that of duplications.

Further work is necessary to better analyze the link between different recombination mechanisms and the type of variants produced. Moreover, SR and PEM approaches were more sensitive for the detection of small variants because of the relatively small size of the insertions (321 bp on average). However, CNVnator, which uses an RD-based approach, is not limited by insert size, but in this study, we were constrained by the criterion that was set, i.e. that only variants predicted by at least two tools were retained. To balance this bias, future studies could use two RD tools to retain large-size variants. This technical bias could partly explain why the number of duplicated regions, which tend to be of larger size than deletions, was small compared to the number of deleted regions.

#### Frequency of variants across animals and breeds

**Fig. 7:**
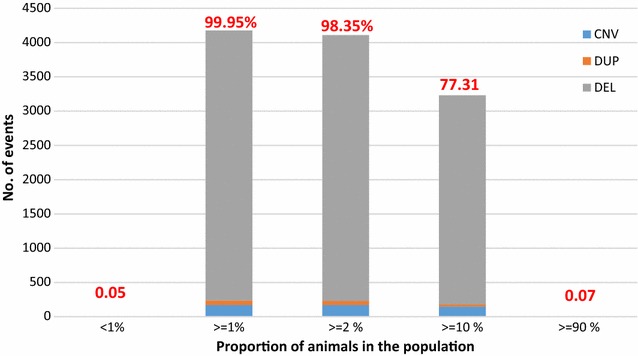
Frequency of variants in the population (N = 200)

The percentage of carriers for each variant varied from 0.5% (1 animal out of 200) to 97% (194 animals out of 200). Overall, 0.05% (two variants) were unique (observed in a single sample) (Fig. 7) and (see Additional file 9: Table S8), which suggests a very recent origin and confirms similar results that were reported in a study on Holstein [46]. The remaining 99.95% of detected variants (4176) were observed in at least 1% of the animals in our panel. These included variants that were shared among several animals within a single breed and variants that likely predated breed formation and were shared by two animal categories (beef and dairy).

Two variants located on BTA17 (CNVR_6167 and CNVR_6267) and a third one located on BTA6 (CNVR_15672) were observed in more than 90% of our population dataset. Analysis of the raw data for each tool revealed that the genome of the remaining 10% of the population also contained these CNV. However, they were discarded from the final results because they were detected with less than three reads, or predicted with only one tool, or did not pass the 70% reciprocal overlap filter. These CNV could also be specific to the individual that was used to produce the UMD3.1 reference sequence. Another explanation could be the presence of locally mis-assembled segments within the reference genome sequence. In addition, CNV prediction based on whole-genome sequences depends strongly on the quality of the bovine reference genome, which is known to be less good than the human reference genome.

The distribution of deletion, duplication, and CNV regions across breeds revealed that only 2.1% were breed-specific, 97.9% were observed in at least two breeds and 17.2% (717) were predicted and validated in the eight breeds analyzed (see Additional file 9: Table S8). Of these 717 variants, 92.5% (663) were deletion-only regions, 6.8% (49) were CNVR and 0.7% (5) were duplication-only regions. Since the UMD3.1 reference genome sequence was obtained from a Hereford animal, these deletion-only and duplication-only regions shared by all breeds could probably be Hereford- or even Dominette-specific events.

The distribution of variants across breeds was highly variable. Almost 96.12% of the variants (4016) were observed in both dairy and beef breeds, 0.17% (7) in only beef and 3.71% (155) in only dairy breeds (see Additional file 9: Table S8). The number of variants shared between breeds did not vary significantly (χ^2^ test, *p* value = 0.26). We observed a small difference in the number of variants detected in dairy breeds only (155) and in beef breeds only (7), which can be partly explained by differences in the number of animals in each breed type and the coverage rate of sequencing used in the Mendelian and twice-sequenced approaches. The 16 pairs and two sire-son-dam trios used in the Mendelian approach were all dairy animals. In the twice-sequenced approach, we explored data from five dairy and nine beef animals, but the coverage rate was much higher for dairy (from 23× to 30× ) than for beef animals (from 9× to 13× ) (see Additional file 1: Table S1).

**Fig. 8:**
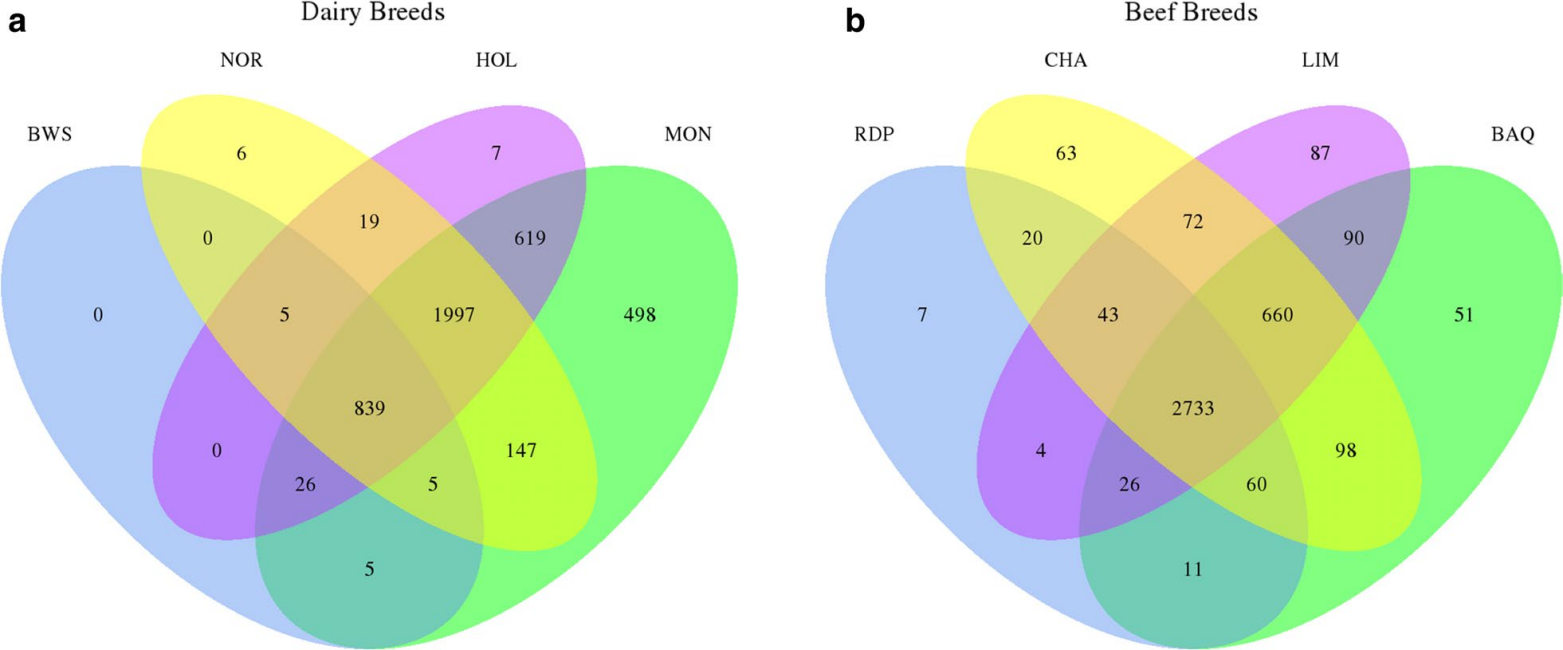
Distribution of variants per breed. **a** Distribution of variants across dairy breeds. **b** Distribution of variants across beef breeds. *BAQ* Blonde d’Aquitaine, *BWS* Brown Swiss, *CHA* Charolaise, *HOL* Holstein, *LIM* Limousine, *MON* Montbéliarde, *NOR* Normande, *RDP* Rouge des Prés

Most of the variants detected in beef breeds were shared by at least three breeds (3522 out of 4023, 86%), while 2872 out of 4171 (69%) were shared by a minimum of three dairy breeds (Fig. 8). The dairy breeds studied here have undergone strong selection to produce the best reproductive animals based on traits of interest; also, artificial insemination is frequently used in dairy breeds to disseminate the selected traits. Thus, each breed has a high degree of specialization, which may explain the small number of variants shared among dairy breeds (69%). On the contrary, the distribution of variants within the beef breeds was very heterogeneous, probably because artificial insemination is much less used in beef breeds.

### Comparison of our dataset with previously described CNV

**Fig. 9:**
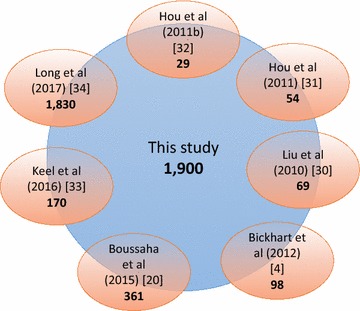
Comparison of predicted variants with published studies. Our results were compared to studies derived from SNP arrays [31, 32], from CGH [30] and from whole-genome sequencing [4, 20, 33, 34]. In this study we analyzed a large panel of breeds (four beef and four dairy breeds) compared to the other studies

We compared all deletion, duplication, and CNV regions identified in our study to publicly available data. Overall, 2278 regions (54.5%) including 2102 deletion regions, 22 duplication regions, and 154 CNVR overlapped with publicly available results from seven published studies (Fig. 9).

Different factors related to the method used to identify CNV probably explain the relatively small proportion of common variants. Among these are the platforms and methods used for CNV calling, and/or the population size and structure of the studied animal populations, and the criteria used to define a CNV region. In a previous study, an array-based CGH approach was used to predict CNV in 90 animals from *Bos taurus*, *Bos indicus* and composite breeds [30] and found no small variants, i.e. the smallest identified CNV was 18,000 bp long. Whereas, in our study, the majority of variants were less than 18,000 bp long. In another study, Hou et al. reported CNV predicted from 472 Angus animals that were genotyped using a medium SNP genotyping array (BovineSNP50) and they did not detect small variants (i.e. mean CNV size = 174,844 bp) [32]. Since we did not include Angus cattle in our study, our results do not contain any Angus-specific CNV. Furthermore, 5.1 and 8.4% of the variants reported by Hou et al. were unique to either an individual or a breed, respectively. This suggests that many bovine CNV are yet to be discovered.

### Functional annotation of CNVR

Analysis of the gene content of deletion-only, duplication-only, and CNV regions revealed that 1100 of all the variants (1000 deletion-only, 21 duplication-only, and 79 CNV regions) identified in our study contained or overlapped with 1803 genes (see Additional file 11: Table S10). Of these, 86% (1577 genes) corresponded to known protein-coding genes, 66 were pseudogenes, 87 genes were small nuclear and nucleolar RNA-coding genes, 26 were microRNA-coding genes, 38 were ribosomal RNAs, and nine were miscellaneous RNA coding genes. Around 81% (1460) of these genes had paralogs. In addition, 231 variants resulted in the deletion of an entire gene, nine in the duplication of an entire gene and 69 CNVR encompassed an entire gene. Furthermore, 186 of the deletion regions removed either partially or entirely a gene, which, in cattle, are described as lacking a paralog; thus, their deletion can alter gene expression and disturb the pathways in which they are involved. Further studies could target homozygous animals to study the effect of these deleted regions.

**Fig. 10:**
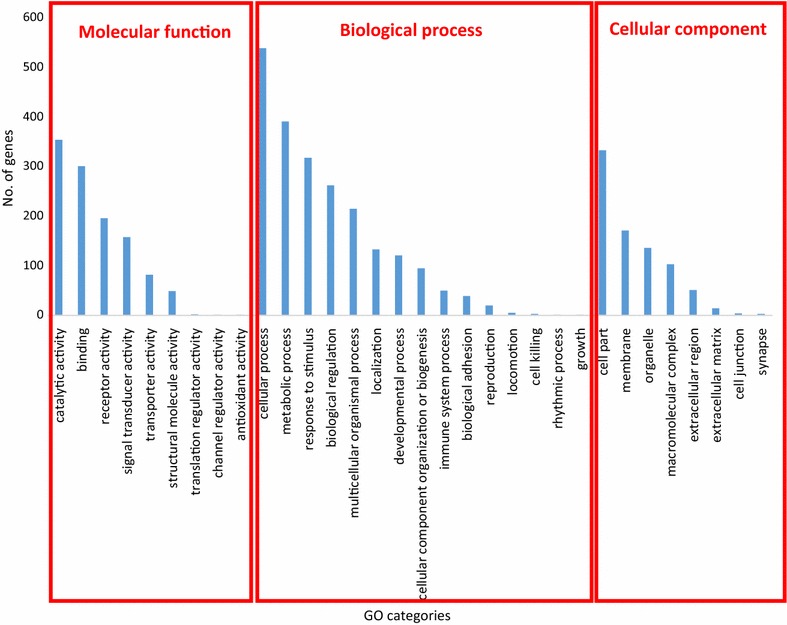
Gene ontology categories of genes impacted by variants

In order to identify the cellular functions associated with genes located in deletions, duplications, and CNVR, we performed a gene ontology (GO) analysis with PANTHER. The GO analysis classified 1442 genes into three GO categories: biological process, cellular component, and molecular function. These genes were enriched in a variety of cellular functions such as cellular and metabolic processes, binding, catalytic capacity, response to stimulus and cell part (Fig. 10). Because this set of genes is involved in a wide range of molecular functions, changes in gene copy number could result in a range of potential phenotypic variations among animals.

The genomic positions of the detected variants were also compared to the positions of publicly available QTL [36]. Overall, 1095 variants overlapped with QTL regions that are associated with milk (10 QTL), production (43 QTL), health (27 QTL), reproduction (26 QTL), or meat and carcass traits (73 QTL) (see Additional file 12: Table S11). In addition, 276 variants overlapped, partially or entirely, with both genes and QTL.

Several of the genes that were found to be located within the variants detected here are known to be associated with several important traits in cattle. One example is the *Bardet*-*Biedl syndrome 7* (*BBS7*) gene, which is partially deleted by the CNVR_15659, has no paralog in the bovine genome and is co-localized with a known bovine QTL for body weight (see Additional file 12: Table S11). This gene is also associated with body weight and male infertility in mouse [47]. We identified this CNVR_15659 region in four dairy animals (one Holstein and three Montbéliarde) and nine beef animals (five Blonde d’Aquitaine, three Charolaise and one Limousine). Additional studies are needed to investigate the link between this deletion and the QTL for body weight.

A second CNVR_12945 region was found to entirely delete the *HSD17B7* (*hydroxysteroid 17*-*beta dehydrogenase 7*) gene, which is related to heifer conception rate trait in Holstein cattle [48]. This region occurred at a higher frequency in beef than in dairy breeds; it was found in only four dairy animals (Montbéliarde) but in 32 beef animals (eight Blonde d’Aquitaine, 11 Charolaise, 10 Limousine and three Rouge des Prés). It would be very interesting to study the effect of this gene on heifer conception rate in the Montbéliarde breed.

Another interesting region, CNVR_10026, which encompasses entirely the *SUPT3H* (*SPT3 homolog*) and *RUNX2* (*runt related transcription factor*) genes, occurred at different frequencies in beef and dairy breeds; it was deleted in 11 dairy animals (four Holstein, six Montbéliarde and one Normande) and 18 beef animals (seven Blonde d’Aquitaine, six Charolaise, four Limousine and one Rouge de Prés). Two more beef animals (one Blonde d’Aquitaine and one Rouge de Prés) had CNVR (both a duplication and deletion in the same region). *SUPT3H* and *RUNX2* are associated with milk fat traits [49]. In addition, we found that *RUNX2* overlaps with a QTL associated with 305-day milk yield and milk protein percentage [50] and *SUPT3H* is upstream of this same QTL.

The duplication CNVR_11771, which we detected in two Montbéliarde individuals, one Charolaise, one Limousine and one Rouge des Prés individual, overlapped with the transcription factor gene *GTF2E2* (*general transcription factor IIE subunit 2*), which is known to be deregulated during *Eimeria bovis* infection [51].

In summary, we found that several of the variants detected in our study could potentially impact genes that are associated with important cattle traits. Future studies are needed to examine these variants in detail, together with phenotypic records, to confirm or infirm their effects.

## Conclusions

Several recently developed NGS-based detection algorithms have led to significant progress in CNV detection. Here, we identified and characterized deletions, duplications, and CNV in eight French cattle breeds. This study represents one of the largest efforts for applying a sequence-based approach to detect CNV in cattle (200 animals). By exploring different complementary approaches and applying a stringent merge strategy, we identified 4178 deletion-only, duplication-only, and CNV regions in both dairy and beef animals. We found 4163 variants by using two in silico approaches (Mendelian inheritance and reproducible predictions from sequences from multiple sequencing runs of the same animal). Of these 4178 variants, 69 were confirmed using SNP genotyping data and 15 other variants were validated using an array-based CGH approach. Our analyses revealed that predictions were most accurate when using a combination of Pindel + CNVnator tools. Some of the variants identified here can potentially affect genes that are involved in economically important cattle traits, and further analyses are necessary to investigate their possible effect. This study will contribute to drawing up a CNV map in French cattle and examining the potential impact of this kind of genetic variation on economically important traits of interest.

## Additional files

**Additional file 1: Table S1.** Number of variants predicted for each individual. This table provides details on each sequenced animal: sequencing coverage, statistics about reads number, its parents if they were sequenced and the total number of predicted variants.

**Additional file 2.** CGH protocol. In this document, a detailed CGH protocol is provided.

**Additional file 3: Table S2.** Variants identified in the 200 whole-genome sequences. This file provides details on all predicted and merged variants before applying validation approaches (in silico and experimental).

**Additional file 4: Table S3.** Variants validated by the Mendelian approach. This table provides details on validated variants using the Mendelian approach and the number of trios, duos or parents with several sons that support the variant.

**Additional file 5: Table S4.** Percentage of validated variants in trios and parent-son pairs. This table includes statistics about the percentage of inherited variants from the parent to its son.

**Additional file 6: Table S5.** Variants validated by the twice-sequenced approach.

**Additional file 7: Table S6.** Variants identified by the array-based CGH approach. This table provides details on variants identified using the array-based CGH approach for 17 animals compared to whole-genome sequencing variants.

**Additional file 8: Table S7.** Selected variants for genotyping on the low-density Bovine BeadChip. In this table, we provide information for validated variants using the low-density Bovine BeadChip. Variants that were validated using the two in silico approaches and the array-based CGH are indicated.

**Additional file 9: Table S8.** Summary of validated variants by the different approaches. This is an inventory of validated variants using the two in silico and/or array-based CGH approaches. This dataset was the final subset of variants retained in this study. Information about breeds and validation approaches that support each variant is indicated.

**Additional file 10: Table S9.** Distribution of variants across chromosomes. This table provides variant counts and chromosome coverage for each bovine autosome.

**Additional file 11: Table S10.** Genes overlapping with identified deletion, duplication and CNV regions. In this table, we report genes that overlap entirely or partially with validated variants.

**Additional file 12: Table S11.** QTL overlapping with identified deletion, duplication and CNV regions. In this table, we report validated variants co-localized with QTL regions and details for each QTL.
