# Supplementary material for: Identification of copy number variation in French dairy and beef breeds using next-generation sequencing
Source: Genet Sel Evol. 2017 Oct 24;49:77. doi: 10.1186/s12711-017-0352-z (PMC5655909; doi:10.1186/s12711-017-0352-z)
Supplement: Supplementary file 2 — Additional file 2. CGH protocol. In this document, a detailed CGH protocol is provided. [file 12711_2017_352_MOESM2_ESM.docx]

**Enzymatic CGH protocol**

1. **Microarray**

The slides used for the experiment were provided by Agilent Technologies: SurePrint G3 Bovine CGH Microarray, 4x180K reference: G4826A-025242.

1. **DNA fragmentation**

As described in the manufacturer’s protocol (Array Based CGH for Genomic DNA Analysis - Agilent), 600ng of genomic DNA (gDNA) was prepared in a maximum volume of 20.2 µL for the control and each sample. An enzyme cocktail composed for one reaction by 2 µl of water, 2.4 µl of 10x restriction enzyme buffer, 0.2 µl of BSA, 0.5 µl of ALU1 and 0.5 µl of RSA1 enzymes was added to each sample of gDNA (control and sample). The digestion was done at 37°C during 2 h, stopped at 65°C during 20 min and kept in ice.

1. **Sample labeling**

5µl of random primers were added to each fragmented DNA (samples and control) and denaturated for 3 min at 98°C and moved on ice. 16 µl of labeling master mix (10 µl of 5x reaction buffer, 5 µL 10x dNTPs and 1 µl of DNA polymerase (Exo (-) Klenow) were added to each sample. Finally, 3 µl of Cyanine 3-dUTP were added in each control preparation and 3 µL of Cyanine 5-dUTP in each sample preparation. Each reaction preparation was incubated at 37°C for 2 h to synthetize fluorescent DNA. The reaction was stopped by increasing the temperature to 65°C for 10 min and the samples were maintained on ice.

1. **Purification of labeled DNA**

The labeled gDNA were centrifuged for 1 min at 6000g to drive the contents off the walls and lid. 430 µl of 1x TE were added to each reaction tube and loaded onto a column to be purified. After 10 min of centrifugation at 14,000g, the flow-through was discarded. 480 µl of 1xTE were added onto each column and centrifuged for 10 min at 14,000g. The flow-through was discarded again. The column was inverted into a new tube and centrifuged for 1 min at 1000g. The final collected volume was around 21 µl per sample and the quality of the labeling was measured on a ND-1000 (Nanodrop_Thermofisher). Finally, the specific activity and the yield of incorporation of labeled Cyanines (Cy3 and Cy5) was calculated.

1. **Hybridization**

As described in the manufacturer’s protocol, the (Array Based CGH for Genomic DNA Analysis - Agilent), we combined 19.5 µl of control and 19.5 µl of sample and added 5 µl of Cot1 DNA, 11 µl of 10x aCGH blocking agent and 55 µl of 2x HI-RPM hybridization buffer. The mix was denatured at 98°C for 3 min and moved to 37°C for 30 min to stabilize DNA. A gasket slide was loaded in the hybridization chamber base and 100 µl of each of four sample preparations were loaded onto the four wells of gasket slide. Then, it was covered by a microarray (as described in §1) and the hybridization chamber was closed tightly by the cover and the clamp. The microarray was incubated at 67°C for 24 h with a rotation at 20 rpm. After hybridization, the microarray was put in the wash buffer I where the two gasket slides were separated. The gasket with samples was moved to a clean wash buffer I and placed on a stirrer for 5 min at room temperature. The slide was washed a third time in the wash buffer 2, which was pre warmed over night at 37°C, for 1 min. The slide was slowly removed from the buffer to minimize droplets and directly scanned to avoid the impact of environmental oxidants.

1. **Microarray scanning**

The microarray was scanned on the MS200 scanner (TECAN) by setting up the laser power at 100 to excite both the Cy3 and Cy5. Tiff images were then generated.

1. **Data analysis**

Quality control of raw data was done with Feature extraction (V11.5.1.1.) from Agilent Technologies and the analyses by using Genomic Workbench (V7.0.4.0.)
